# Chromosomal abnormalities and copy number variations in fetuses with ventriculomegaly: a multicenter retrospective study

**DOI:** 10.1186/s13039-026-00768-1

**Published:** 2026-05-27

**Authors:** Anh Huu Duc Nguyen, Phan Duc Tran, Phuc Hong Dinh, Trang Thi Dao, Ngoc Thi Minh Nguyen, Anh Thi Lan Luong, Lan Thi Ngoc Hoang, Phuong Thi Kim Doan, Hung Trong Mai, Linh Thuy Dinh, Vuong The Pham, Giang Thi Thu Phan, Thang Duc Bui, Linh Thuy Tran, Cuong Danh Tran

**Affiliations:** 1 Hanoi Medical University, Hanoi, Vietnam; 2 Phenikaa University, Hanoi, Vietnam; 3 Hanoi Obstetrics and Gynecology Hospital, Hanoi, Vietnam; 4 National Hospital of Obstetrics and Gynecology, Hanoi, Vietnam; 5 Hanoi Medical University Hospital, Hanoi, Vietnam

**Keywords:** Fetuses with ventriculomegaly (VM), Chromosomal abnormalities, Copy number variations

## Abstract

**Background:**

This study aimed to analyze chromosomal characteristics and copy number variations (CNVs) in fetuses with ventriculomegaly (VM), thereby comparing genetic features between the isolated VM group and the non-isolated VM. Simultaneously evaluate the efficacy of karyotyping and CNV analysis in prenatal diagnosis of fetuses with VM.

**Methods:**

This retrospective cross-sectional study included 78 fetuses diagnosed with VM at the Prenatal Diagnosis Center, National Hospital of Obstetrics and Gynecology, the Clinical Genetics Center, Hanoi Medical University Hospital, and the Prenatal Diagnosis Center, Hanoi Obstetrics and Gynecology Hospital from January 2023 to December 2024. Genetic testing methods included conventional karyotyping and CNV analysis.

**Results:**

Chromosomal abnormalities were detected in 14.1% (11/78) of cases by karyotyping, while CNVs were identified in 17.9% (14/78). CNVs were significantly more frequent in the non-isolated VM group compared to the isolated group (29.4% vs. 9.1%, *p* = 0.02). Both methods concordantly detected six cases of aneuploidy. Three cases of microdeletions/duplications were detected by CNV analysis, while karyotyping revealed no abnormalities. An additional four cases with chromosomal structural abnormalities detected by karyotyping had their deletion/duplication segments precisely characterized for size and location through CNV analysis.

**Conclusion:**

Genetic testing using amniotic fluid samples should be considered an essential component in the prenatal diagnostic approach to VM. Non-isolated VMs exhibit significantly higher CNV risk than isolated VMs. CNV analysis enhances diagnostic efficacy for genetic abnormalities compared to karyotyping, while simultaneously facilitating genetic variant classification, thereby providing more detailed information for prognostic assessment. The combined approach of karyotyping and CNV analysis improves the detection rate of genetic abnormalities, supporting enhanced genetic counseling and superior pregnancy management.

## Background

Ventriculomegaly (VM) is frequently detected fetal central nervous system anomalies during prenatal ultrasound, defined as dilation of the lateral cerebral ventricles exceeding 10 mm [1]. It is associated with a broad spectrum of etiologies, including chromosomal abnormalities, copy number variations (CNVs), congenital infections, and structural malformations, making prenatal prognosis highly variable [2]. Conventional karyotyping has long been the standard method for detecting chromosomal abnormalities in fetuses with VM. However, this approach is limited in its ability to identify submicroscopic genomic imbalances. In recent years, CNV analysis using chromosomal microarray techniques has emerged as a high-resolution tool capable of detecting clinically significant deletions and duplications that are not visible on karyotyping. Previous studies have demonstrated the diagnostic value of CNV analysis in fetuses with central nervous system anomalies, including VM [3, 4]. However, data from Southeast Asian populations, particularly Vietnam, remain limited. In addition, the clinical significance of genetic findings may differ between isolated VM and VM associated with other structural abnormalities, a distinction that remains incompletely characterized. Therefore, this study aimed to [1] investigate the prevalence and distribution of chromosomal abnormalities and CNVs in fetuses with VM, with a comparison between isolated and non-isolated cases; and [2] assess the incremental diagnostic value of CNV analysis over conventional karyotyping.

## Methods

### Design and setting of the study

This retrospective study included 78 fetuses diagnosed with ventriculomegaly (VM) at the Prenatal Diagnosis Center, National Hospital of Obstetrics and Gynecology, Clinical Genetics Center, Hanoi Medical University Hospital, and Prenatal Diagnosis Center, Hanoi Obstetrics and Gynecology Hospital, Hanoi, Vietnam from January 2023 to December 2024. Genetic testing methods included conventional karyotyping and CNV analysis.

The study population was divided into two groups: isolated ventriculomegaly and non-isolated ventriculomegaly.

Isolated ventriculomegaly was defined as ventriculomegaly without any additional structural abnormalities detected on prenatal ultrasound. Non-isolated ventriculomegaly was defined as ventriculomegaly associated with one or more additional structural anomalies, including both central nervous system and extra-central nervous system abnormalities. Prenatal diagnosis was performed via amniocentesis. Amniotic fluid cells were cultured and subjected to genetic analysis, including conventional karyotyping and/or CNV analysis.

### Genetic testing procedures

#### Sample collection and DNA extraction

Amniotic fluid samples (15–20 mL) were obtained via ultrasound-guided amniocentesis. Each sample was divided for conventional cytogenetic analysis and genomic DNA extraction. Genomic DNA was extracted using commercially available kits (QIAamp DNA Mini Kit, Qiagen, Germany) according to the manufacturer’s instructions.

#### Karyotyping

Conventional karyotyping was performed on cultured amniocytes using standard G-banding techniques [5]. At least 20 metaphase cells were counted, and five metaphase cells were examined to detect numerical and structural chromosomal abnormalities.

#### CNV

In this study, CNV analysis was performed using chromosomal microarray analysis (CMA) or copy number variation sequencing (CNV-seq), according to the protocols of the participating centers.

For CMA, genomic DNA was analyzed using either array comparative genomic hybridization (array CGH) or single-nucleotide polymorphism (SNP) array platforms. Specifically, array CGH was performed using the SurePrint G3 ISCA V2 CGH 8 × 60 K microarray (Agilent Technologies, USA), and SNP array analysis was performed using the CytoScan 750 K platform (Thermo Fisher Scientific, USA). Hybridization, washing, and scanning were carried out according to the manufacturers’ protocols using the SureScan Microarray Scanner (Agilent Technologies). Data were analyzed using CytoGenomics software (Agilent) or Chromosome Analysis Suite (Thermo Fisher Scientific), with reference genomes GRCh37/hg19 or hg38.

For CNV-seq, genomic DNA libraries were prepared using standard protocols (e.g., NEBNext kits), followed by low-pass whole-genome sequencing on an Illumina platform (e.g., NextSeq). Sequencing reads were aligned to the human reference genome, and CNVs were identified based on read-depth analysis across genomic bins (~ 100 kb resolution).

The detection threshold for CNVs was approximately 400 kb. Balanced chromosomal rearrangements, point mutations, and low-level mosaicism below this resolution were not reliably detected.

The clinical significance of the detected CNVs was interpreted based on information in databases and academic publications. Based on guidelines of the American College of Medical Genetics and ClinGen, the CNVs in this study were classified into pathogenic (P), likely pathogenic (LP), benign (B), likely benign (LB), or variants of uncertain significance (VUS) [6].

### Statistical analysis

The statistical analysis was performed using STATA version 18.0 (StataCorp LLC, College Station, TX, USA). Continuous variables are reported as the mean ± standard deviation (SD). Variables were compared using the T-test or Fisher’s exact test. P-values < 0.05 were considered statistically significant.

## Results

### Chromosomal abnormalities and CNVs detected in fetuses with ventriculomegaly

A total of 78 fetuses with ventriculomegaly (VM) were included, comprising 44 cases of isolated VM (56.4%) and 34 cases of non-isolated VM (43.6%). The baseline characteristics of the study population are summarized in Table 1. The mean maternal age was 28.53 ± 5.17 years, and the mean gestational age was 21.79 ± 3.55 weeks.

**Table 1:** Characteristics of fetuses with ventriculomegaly (*n* = 78)

Characteristic	Isolated ventriculomegaly	Non-isolated ventriculomegaly	Total	*p*
Patients, n (%)	44 (56.4%)	34 (43.6%)	78 (100.0%)	**-**
Maternal age (years)				
o < 35	39 (88.6%)	29 (85.3%)	78 (100.0%)	0.66
o ≥ 35	5 (11.4%)	5 (14.7%)		
Mean maternal age (SD)	28.59 ± 5.35	28.44 ± 5.08	28.53 ± 5.17 (17–41 years)	0.34
Mean gestational age (weeks)	21.94 ± 3.67	21.50 ± 3.44	21.79 ± 3.55 (16–32 weeks)	0.59

T-test, Chi-square test

As shown in Table 2, the detection rate of chromosomal abnormalities by karyotyping was 14.1% (11/78), with no significant difference between isolated and non-isolated VM groups (*p* > 0.05).

**Table 2:** Chromosomal abnormalities in fetuses with ventriculomegaly detected by karyotyping

Karyotyping	Isolated ventriculomegaly	Non-isolated ventriculomegaly	Total	*p*
Normal	40 (90.9)	27 (79.4)	67 (85.9)	0.19
Abnormalities	4 (9.1)	7 (20.6)	11 (14.1)	
**Total**	**44 (100.0)**	**34 (100.0)**	**78 (100.0)**	

Fisher-exact test

CNV analysis identified chromosomal abnormalities in 17.9% (14/78) of cases (Table 3). The detection rate was significantly higher in non-isolated VMs than in isolated VMs (29.4% vs. 9.1%, *p* = 0.02).

**Table 3:** Chromosomal abnormalities in fetuses with ventriculomegaly detected by CNV analysis

CNV	Isolated ventriculomegaly	Non-isolated ventriculomegaly	Total	*p*
Normal	40 (90.9)	24 (70.6)	64 (82.1)	0.02*
Abnormalities	4 (9.1)	10 (29.4)	14 (17.9)	
**Total**	**44 (100.0)**	**34 (100.0)**	**78 (100.0)**	

Fisher-exact test, *p < 0.05

Two cases of sex chromosome aneuploidies (47,XXY and 47,XYY) were identified. When excluding these cases, the adjusted diagnostic yield was 9/78 (11.5%) for karyotyping and 12/78 (15.4%) for CNV analysis.

### Comparison between karyotyping and CNV analysis

The concordance between karyotyping and CNV analysis is presented in Table 4. Both methods consistently detected all six cases of aneuploidy, including trisomy 21 (*n* = 4), 47,XYY (*n* = 1), and 47,XXY (*n* = 1). In addition, one case of mosaic trisomy 9 detected by karyotyping was confirmed by CNV analysis. CNV analysis provided additional diagnostic information beyond karyotyping. Three pathogenic submicroscopic abnormalities were detected exclusively by CNV analysis.

**Table 4:** Consistency analysis of karyotyping and CNV analysis

Abnormality detection^α^		
Karyotyping	CNV	n, %
Negative (–)	Negative (–)	64, 82.05
Negative (–)	Positive (+)	3, 3.85
Positive (+)	Positive (+)	11, 14.1

^a^Positive: chromosomal abnormality detected; Negative: no abnormality detected

Three cases with pathogenic copy number variants (CNVs) not detectable by karyotyping. These include VN032,VN037, VN073. Furthermore, detailed diagnostic comparison between karyotyping and CNV analysis is presented in Table 5.

**Table 5:** Diagnostic comparison between karyotyping and CNV analysis in ventriculomegaly

ID case	MA	GA	Abnormalities	Karyotype	CNV			
					Variant	Type	Size	C
VN026	36	25w4d	Ventriculomegaly Cleft lip and palate Persistent left superior vena cava Aberrant right subclavian artery	47,XX,+9[73]/46,XX[5]	arr[GRCh38]9p24.3p11.2(208455_40937364)x4[0.6]	Gain	40.7 Mb	LP
VN032	25	19w3d	Ventriculomegaly	46,XY, der(2)t(2;5)(q37,p13)	arr[GRCh38]2q37.3(239852434_241840106)x1 arr[GRCh38]5p15.33p13.3(113462_29030539)x3	Loss Gain	1.98 Mb 28 Mb	P P
VN037	20	25w	Joubert syndrome	46,U	del(17)(p12)(chr17:g.14,196,683 − 15,496,686)	Loss	1.3 Mb	P
VN038	25	19w	Ventriculomegaly Absent nasal bone	46,XY, rec(5)del(5pterp15.2) dup(5p15.2p13)	del(5)(p15.33-p15.2)(chr5:g.99,885-9,899,888) dup(5)(p15.2-p13.2)(chr5:g.9,899,888 − 34,499,895)	Loss Gain	9.8 Mb 24 Mb	P P
VN041	40	21w	Ventriculomegaly Absent nasal bone	47,XX,+21	Trisomy 21			P
VN044	27	17w	Ventriculomegaly	47,XX,+21	Trisomy 21			P
VN047	23	25w	Ventriculomegaly	47,XYY	47,XYY			P
VN049	27	18w	Ventriculomegaly	47,XXY	47,XXY			P
VN050	22	21w2d	Ventriculomegaly Short and thin corpus callosum	46,U	arr[GRCh37] 6q26(161453689_164462426)x3, arr[GRCh37] 6q27(164514705_170911240)x1 liftOver GRCh38: 6q26(161032657–164041394)x3 6q27(164093673–170602152)x1	Gain Loss	3 Mb 6.3 Mb 3 Mb 6.5 Mb	VUS P
VN055	33	23w1d	Dandy–Walker malformation	46,U	del(14)(q11.2)(chr14:g.21,831,844 − 22,531,050)	Loss	0.7 Mb	B
VN061	32	25w	Hemimegalencephaly	47,XXY	47,XXY			P
VN065	28	16w3d	Ventriculomegaly Polydactyly of the right hand	47,XY,+21	Trisomy 21			P
VN073	29	17w	Ventriculomegaly Fallot IV	46,XX, del(8)(p21)	del(8)(p21.2-p11.21)(chr8:g.23,642,487 − 42,142,482)	Loss	18.5 Mb	P
VN075	34	17w	Ventriculomegaly Nasal bone hypoplasia	47,XY,+21	Trisomy 21			P

MA, maternal age; GA, gestational age; pathogenic (P); benign (B); variants of uncertain significance (VUS); C, Classfication

## Discussion

In this multicenter study, we demonstrated that CNV analysis provides a higher diagnostic yield than conventional karyotyping in fetuses with ventriculomegaly (VM), particularly in non-isolated cases. These findings are consistent with the results of previous studies [7].

The prevalence of CNVs was significantly higher in non-isolated VM compared to isolated VM, highlighting the importance of phenotypic stratification in prenatal genetic evaluation.

In our study, the detection rate of chromosomal abnormalities by conventional karyotyping was consistent with previously reported rates in fetuses with the central nervous system [2, 8], supporting its continued role as a fundamental component of prenatal genetic evaluation. The CNV detection rate in our cohort was also comparable to previous studies [9, 10], reinforcing the growing evidence that submicroscopic genomic imbalances play a significant role in the etiology of ventriculomegaly and highlighting the added diagnostic value of high-resolution genomic testing.

Our findings demonstrate the incremental diagnostic value of CNV analysis over conventional karyotyping. While both methods showed complete concordance in detecting aneuploidies, CNV analysis identified additional submicroscopic abnormalities that were not detectable by karyotyping. Furthermore, CNV analysis enabled more precise characterization of structural chromosomal abnormalities, thereby improving genotype phenotype correlation and supporting more accurate prenatal counseling.

Regarding the detection of aneuploidies, sex chromosome aneuploidies (47,XXY and 47,XYY) were identified in our cohort. While these chromosomal anomalies are definitively classified as pathogenic genomic findings that require extensive genetic counseling, they are not commonly recognized as causative factors for ventriculomegaly [1]. Therefore, their co-occurrence with VM in this study is likely incidental rather than causal [11]. This distinction is crucial when interpreting the true diagnostic yield of prenatal genetic testing; while reporting all pathogenic findings is mandatory for patient management, failing to stratify these incidental findings may overestimate the proportion of clinically relevant abnormalities directly responsible for the VM phenotype.

The significantly higher prevalence of CNVs in non-isolated ventriculomegaly suggests that the presence of additional structural anomalies may indicate a higher likelihood of underlying genomic imbalance. From a clinical perspective, this finding supports the use of more comprehensive genetic testing strategies, particularly in non-isolated VM cases.

Representative cases in our cohort further illustrate the clinical utility of CNV analysis. Submicroscopic deletions and duplications undetectable by karyotyping were identified (cases VN037, VN073), and complex structural rearrangements were more precisely characterized (cases VN026, VN032). In addition, incidental findings and variants of uncertain significance (case VN050) highlight the challenges of interpretation and the need for careful genetic counseling. In this case, the coexistence of a 6q26 gain and a 6q27 loss may suggest a complex structural rearrangement, such as an inverted duplication with terminal deletion (inv-dup-del). However, no confirmatory analyses, such as fluorescence in situ hybridization (FISH), were performed; therefore, the precise genomic architecture and underlying mechanism could not be fully elucidated.

Importantly, a normal karyotype or the absence of pathogenic CNVs does not exclude the presence of underlying genetic disorders. Single-gene conditions, including X-linked disorders such as L1CAM-related hydrocephalus, may not be detectable using karyotyping or CNV analysis. In a similar context, Joubert syndrome is typically caused by pathogenic variants in genes involved in ciliary function rather than chromosomal imbalances. This highlights the complementary role of next-generation sequencing approaches, such as whole-exome sequencing, particularly in cases with abnormal fetal imaging but negative CNV results.

These findings also suggest that additional genomic approaches, such as next-generation sequencing, may be warranted in selected cases where no pathogenic CNVs are identified despite abnormal fetal imaging. This study contributes novel data from a Vietnamese population, addressing the limited evidence from Southeast Asia. Given potential differences in genetic background and clinical practice, these findings provide valuable regional insights into the genetic evaluation of ventriculomegaly.

### Strengths and limitations

A major strength of this study is its multicenter design, which provides a more representative sample of the Vietnamese population. Furthermore, the combined use of conventional karyotyping and CNV analysis allowed for a direct evaluation of their incremental diagnostic value. However, there were some limitations, such as a relatively small sample size, and postnatal follow-up data were not available to assess long-term outcomes. In addition, while CNV-seq was utilized for detecting structural variants, high-depth next-generation sequencing approaches, such as Whole Exome Sequencing (WES) or targeted gene panels, were not routinely performed, which may have limited the detection of single-gene disorders. Confirmatory techniques such as fluorescence in situ hybridization (FISH) or other orthogonal validation methods were not performed. As a result, the underlying structural mechanisms of complex CNVs, such as potential inverted duplication with terminal deletion (inv-dup-del) events, could not be further characterized. Future studies with larger cohorts and longitudinal follow-up are needed to further refine the genetic assessment of VM.

## Conclusion

The proportion of chromosomal abnormalities and CNVs in fetuses with VM was high. Therefore, genetic testing using amniotic fluid samples should be considered an essential component in the prenatal diagnostic approach to VM. Non-isolated VM cases were found to have a significantly higher risk of pathogenic CNVs than isolated VM cases, highlighting the relevance of risk stratification based on fetal morphological features for guiding appropriate genetic testing strategies. The combined use of karyotyping and CNV analysis offers complementary diagnostic benefits and enhances the overall effectiveness of prenatal evaluation and decision-making in pregnancies complicated by suspected central nervous system anomalies such as VM.

## Data Availability

The datasets generated and/or analyzed during the current study are not publicly available due to institutional regulations and patient confidentiality policies.
